# Impacts of counseling on knowledge, attitude and practice of medication use during pregnancy

**DOI:** 10.1186/s12884-017-1316-6

**Published:** 2017-04-27

**Authors:** Ramesh Devkota, G. M. Khan, Kadir Alam, Binaya Sapkota, Deepa Devkota

**Affiliations:** 1 Department of Drug Administration, Kathmandu, Nepal; 2 Pokhara University, Kaski, Nepal; 3 B.P. Koirala Institute of Health Sciences, Dharan, Nepal; 4 Nobel College, Kathmandu, Nepal; 5 People’s Dental College and Hospital, Kathmandu, Nepal

**Keywords:** Impacts, Counseling, Medication, Pregnancy, KAP, Nepal

## Abstract

**Background:**

Counseling has a significant role in improving knowledge, attitude and practice outcomes of pregnant women towards medication use. Proper counseling thus could be beneficial to prevent any medication related misadventure during pregnancy. The present study was aimed to assess the knowledge, attitude and practice (KAP) of pregnant women towards their medications, to provide counseling regarding their understanding of medication use during pregnancy and evaluate the impacts of such counseling.

**Methods:**

Pre- post interventional (counseling) study was conducted at Manipal Teaching Hospital, Nepal among pregnant women who presented with complication and were prescribed at least one medication. A total of 275 pregnant women were included in the study. A structured questionnaire was used to assess the knowledge, attitude and practice of pregnant women before and after counseling. The impacts of counseling were then evaluated using suitable statistical methods.

**Results:**

Of the total participants 229 completed the post counseling survey. Majority of the participants were in the age group 20-24 (43.2%), primigravida (59.4%) and in third trimester (58.6%). Housewives comprised 61.1% of participants and majority had received a University degree (33.2%).

The mean and median scores assessed before counseling showed that there was no significant difference in the KAP scores with respect to age, trimester and gravidity whereas KAP scores with respect to occupation and level of education were statistically significant. There was an increase in mean and median KAP scores after counseling and the impacts of counseling was found to be statistically significant (*p* = <0.001).

**Conclusion:**

Counseling had a positive impact on knowledge, attitude and practice of pregnant women towards medication and thus it could be considered a suitable method to encourage safe medication during pregnancy.

**Electronic supplementary material:**

The online version of this article (doi:10.1186/s12884-017-1316-6) contains supplementary material, which is available to authorized users.

## Background

Patient counseling has always been considered as one of the effective measures to enhance medication adherence. In the absence of proper counseling, patients may not have enough information about their medication, including the dosage regimen, side effects or a missed dose. Lack of information may compel them to not take the medication in the way it was intended, which in turn may result in therapeutic failure, adverse effects, additional expenditure on investigations and treatment, and even hospitalization [1]. The choice of a medicine during pregnancy is even more difficult, since some medicines may have serious side effects on the fetus. The potential effects of a medicine on the fetus should always be considered and medication regimen during pregnancy should be chosen in such a way that it maximizes the effectiveness while minimizing the maternal and fetal risk [2]. Thus, healthcare providers, who treat pregnant women, should be familiar with the medicine and modalities of their use for providing optimal therapeutic services [3]. Besides prescription medicines, pregnant women may also take OTC (over the counter) medicines which need to be identified and addressed accordingly [4]. Both the physicians and pharmacists have an important role in making the pregnant women know about their conditions, any complications they have during pregnancy and the medications they are taking.

Studies reveal that pregnant women often take medicines without sufficient knowledge [5, 6]. Self medication habits among the pregnant women have been found to be common in many developing countries [7] and many of them might not know the reason of taking a medication [8]. The KAP (knowledge, attitude and practice) regarding nutrition and other health care facilities may also play a significant role in safe motherhood [9–11]. Lack of proper medication knowledge and practice among pregnant women might eventually have serious impacts on health of both the mother and child. Thus, effective interventions are required to enhance knowledge, attitude and practice of pregnant women regarding safe medication during pregnancy.

Education, access to health care facilities, availability of health care personnel, transportation and economy are major determinants of maternal health in Nepal which have posed difficulties in availing healthcare facilities during pregnancy [12]. These variables have led to more homebirths (63% of total births in 2011) than in a health care facility [12]. Likewise the literacy rate of Nepal as per census of 2011 is 64% with female literacy rate only 58% [13]. The GNI (Gross National Income) per capita of Nepal is only 700 USD [14] which leads to economic barriers to access healthcare facilities and medications. Less economy might lead to self medication [15] which could be a wrong choice during pregnancy. All these factors impart that pregnant women in Nepal might not be receiving and or have the medication knowledge that they should.

Health care facilities should ensure that patients receive sufficient knowledge about their medications before leaving the facility. Counseling is one of the suitable methods to impart this required knowledge. Effective counseling has been associated with better and positive outcomes in terms of knowledge, attitude and practice of the safe and effective utilization of medicines during pregnancy [3, 16–18]. Thus, to enhance medication safety during pregnancy, this research was aimed to assess the knowledge, attitude and practice of pregnant women towards their medications, to provide counseling regarding their understanding of medication use during pregnancy and evaluate the impacts of such counseling.

## Methods

### Study design, site and sample size

Hospital based, pre-post interventional study was conducted at OBG (Obstetrics and Gynecology)Ward of Manipal Teaching Hospital, Fulbari, Kaski, Nepal from September 2013 to February 2014. A total of 275 women who met the inclusion criteria were included in the study.

### Sampling technique

Simple purposive sampling technique was applied.

### Inclusion criteria

- Pregnant women who came for antenatal care at the OBG ward of the study site
- Who presented with at least one complication and were prescribed at least one medicine
- Patients only in the OPD (Out Patient Department) section

### Exclusion criteria

- Pregnant women who did not have any complications were excluded from the study.
- Patients who were admitted to the hospital

### Data collection and pilot testing

Data was collected using a structured questionnaire consisting of 15 questions subdivided into 7 related to knowledge and 4 each for attitude and practice. For knowledge section, each question was provided with three alternatives and scores were given as: yes = 2, no = 0, uncertain = 1. The maximum total score for this section was 14. Five-point likert scale was used for accessing attitude and practice scores and scoring was given as: strongly agree = 5, agree = 4, uncertain = 3, disagree = 2, strongly disagree = 1. The maximum total score for these sections were 20 each. Thus, the maximum total score for the questionnaire was 54.

The questionnaire was developed with reference to relevant literature reviews and expert consultation to match the scope of the study. The initially drafted questionnaire was distributed to the pharmacy experts, professors and OBG physicians of the hospital and their opinions and suggestions regarding the content, relevance, and appropriateness of the items in the questionnaire were utilized to prepare the final version (shown in Additional file 1). This questionnaire was also converted to the local Nepali language. Initial pilot study was conducted in 20 pregnant patients for pre-testing of the questionnaire with all the KAP questions. The collected responses were analyzed for the content validity with SPSS. Overall Cronbach’s alpha value of 0.857 showed that the questionnaire was reliable [19].

The questionnaire was distributed to participants after they received their medicines from the hospital pharmacy. The purpose and nature of the study was clearly explained to every individual (and their parents/spouse wherever applicable). The participation was voluntary and written informed consent was obtained from all the participants prior to data collection. After collecting the demographic data, the participants were asked to tick their most appropriate response with respect to each question and illiterate were assisted verbally. Based upon their responses the participants were then counseled regarding proper medication during pregnancy. Educational package for the pregnant included verbal counseling and face to face interaction. The follow up date of the patients were noted.

The same questionnaire was distributed again to the participants when they came for follow up. Their responses were measured before their visit to the physician. All patients were waited on their voluntary follow-up and no attempt was made to call the patients that did not show up during their specified follow-up time given by the physician. The maximum waiting time for the participants was arbitrarily set to ten days beyond the allocated follow up date. This was to make sure that participants might have some unforseen circumstances which prevented them to make up at the hospital on their allocated date and time. The collected data before and after counseling were then analyzed.

### Statistical analysis

Demographic characteristics of participants including age, trimester, gravidity, occupation and level of knowledge were analyzed with the descriptive statistics using mean, standard deviation, median, range, frequency and percentage. The association of knowledge, attitude and practice scores with relation to demographics was analyzed using Kruskal Wallis test and the impacts of counseling was measured by Wilcoxon Rank Test. Microsoft Excel 2007 and SPSS version 22 were used for the data analysis.

## Results

Of the total 275 participants, 229 completed the post counseling survey. Remaining 46 were lost to follow-up due to unknown reasons. So, results have been presented with reference to the data of those 229 participants who completed the study.

### Demographic details

Majority of the patients in the study were from the age group 20-24 (43.2%). Of the study population, 58.6% were from third trimester and 59.4% were primigravida. More than half of the women were housewives (61.1%) and majority of the women had a University degree. The demographic detail is shown in Table 1.

**Table 1:** Demographic characteristics of study subjects

Category	Frequency (Percentage)
Age group	
15-19	24 (10.5%)
20-24	99 (43.2%)
25-29	80 (34.9%)
30-34	23 (10%)
35-39	2 (0.9%)
40-44	1 (0.4%)
Trimester	
First	38 (16.6%)
Second	61 (26.6%)
Third	130 (58.6%)
Gravidity	
Primigravida	136 (59.4%)
Multigravida	93 (40.6%)
Occupation	
Housewife	140 (61.1%)
Skilled labor	35 (15.3%)
Unskilled labor	13 (5.7)
Student	34 (14.8%)
Business	7 (3.1%)
Educational Status	
Illiterate	6 (2.6%)
Primary	26 (11.4%)
Secondary	64 (27.9%)
Higher secondary	57 (24.9%)
University degree	76 (33.2%)

### Assessment of knowledge, attitude and practice of the participants towards medication use during pregnancy before counseling

It was seen that before counseling 68.1% of the patients knew about their complications, 46.3% could tell the name of medicine prescribed, 21.8% had knowledge of the use of prescribed medicines, 48.9% knew that medicines could cause adverse effects, 65.9% knew all medicines might not be safe during pregnancy, 71.6% had a knowledge that unnecessary medicines could harm mother and fetus and 30.6% knew that unwanted medication during pregnancy could affect fetal organogenesis and development. With respect to attitude questions, 75.6% believed that they should ask about their complications and medicines with physicians or pharmacists, 67.3% agreed that they should immediately notify any adverse effects to physician, nurse or pharmacist, 62% believed that they should not take unnecessary medications during pregnancy and 75.9% had a positive attitude that unnecessary harms due to medications can be minimized by consultation with health care professionals. Likewise, in relation to medication practice habit it was seen that 64.2% of the patients had a habit of self medication, 75.5% had changed this habit after being pregnant. Only 10.3% of the respondents had a habit of asking about safety of medication during pregnancy and 54.6% used to take medications exactly as prescribed (proper time, duration, refill, etc.). The responses to individual questions are detailed in Additional file 2. The knowledge, attitude and practice scores of the respondents before counseling are presented in Table 2.

**Table 2:** Knowledge, Attitude and Practice scores before counseling

Category	Mean ± SD	Median (Range)	Minimum-maximum score
Knowledge	8.8 ± 3.6	10 (14)	0-14
Attitude	15.2 ± 1.9	15 (10)	10-20
Practice	11.8 ± 2.6	12 (13)	5-18

Kruskal Wallis test was run at 95% confidence interval to see the difference in baseline (before counseling) knowledge, attitude and practice scores of pregnant women with respect to their demographic variables. It was seen that there was no significant difference in the baseline KAP scores with respect to age, trimester and gravidity whereas KAP scores with respect to occupation and level of education were statistically significant as presented in Table 3, Table 4 and Table 5.

**Table 3:** Baseline knowledge score mean rank according to demographic category

Category	Baseline knowledge score mean rank	*p* value
Age group		
15-19	100.42	0.524
20-24	111.26	
25-29	125.65	
30-34	110.24	
35-39	125.50	
40-44	72.00	
Trimester		
First	121.29	0.770
Second	116.03	
Third	112.68	
Gravidity		
Primigravida	113.85	0.749
Multigravida	116.68	
Occupation		
Housewife	104.15	0.007
Skilled labor	144.34	
Unskilled labor	104.81	
Student	134.26	
Business	110.57	
Educational status		
Illiterate	17.58	<0.001
Primary	61.83	
Secondary	90.32	
Higher secondary	120.28	
University degree	157.70

**Table 4:** Baseline attitude score mean rank according to demographic category

Category	Baseline attitude score mean rank	*p* value
Age group		
15-19	103.48	0.526
20-24	116.33	
25-29	119.64	
30-34	102.46	
35-39	171.75	
40-44	64	
Trimester		
First	124.62	0.604
Second	114.06	
Third	112.63	
Gravidity		
Primigravida	118.57	0.317
Multigravida	109.78	
Occupation		
Housewife	100.97	<0.001
Skilled labor	154.70	
Unskilled labor	96.23	
Student	135.91	
Business	130.36	
Educational Status		
Illiterate	60.50	<0.001
Primary	53.08	
Secondary	88.78	
Higher secondary	110.16	
University degree	166.20

**Table 5:** Baseline practice score mean score according to demographic category

Category	Baseline practice score mean rank	*p* value
Age group		
15-19	113.29	0.600
20-24	110.50	
25-29	124.44	
30-34	107.41	
35-39	63.75	
40-44	123.00	
Trimester		
First	137.50	0.070
Second	110.44	
Third	110.56	
Gravidity		
Primigravida	118.22	0.371
Multigravida	110.30	
Occupation		
Housewife	104.61	0.011
Skilled labor	143.51	
Unskilled labor	102.19	
Student	132.53	
Business	118.79	
Educational Status		
Illiterate	25.50	<0.001
Primary	67.87	
Secondary	101.65	
Higher secondary	117.81	
University degree	147.33	

### Evaluation of the impact of counseling

After counseling it was seen that there was significant increase in the knowledge, attitude and practice scores of the respondents. When asked on follow-up visits after initial counseling, 91.3% knew about their complication, 69.9% could tell name of medicines prescribed, 46.3% elaborated the use of medicines and 93%, 100%, 99.1% and 95.6% successively replied that medicines can show adverse effects, all medicines might not be safe during pregnancy, unnecessary medicines during pregnancy can affect health of mother and fetus and unwanted medications during pregnancy can affect fetal organogenesis and development. Similarly, with respect to their attitude, 93.9%, 95.2%, 98.7% and 97.4% respectively believed that they should ask about their complication and medication, notify any drug related adverse effects, not take unnecessary medicines during pregnancy, and potential medication related harms can be prevented by consultation with health care professionals. As with medication practice habit, after counseling percentage of respondents taking medicines without consultation significantly reduced to 2.2%, 90.9% changed their medication taking habits, 25.3% started asking about medication safety during pregnancy and 88.7% adhered to proper medicine use. The detailed responses after counseling are presented in Additional file 2. The knowledge, attitude and practice scores after counseling are summarized in Table 6.

**Table 6:** KAP scores after counseling

Category	Mean ± SD	Median (Range)	Minimum-maximum score
Knowledge	12.86 ± 1.27	13(7)	7-14
Attitude	17.81 ± 1.55	18(6)	14-20
Practice	15.96 ± 2.05	16 (11)	9-20

The KAP scores before and after counseling were statistically tested by Wilcoxon rank test at 95% confidence interval to evaluate the impacts of counseling on knowledge, attitude and practice of the respondents. It was seen that there was a significant association between counseling and its impact on knowledge, attitude and practice scores of the patients (*p* = <0.001).

## Discussion

The KAP survey showed that majority of the pregnant women possessed positive attitude towards medication use during pregnancy as compared to the percentage responses for knowledge and practice. More than 50% of the participants knew about their complications before counseling but fewer had knowledge about the medicines prescribed and their uses. Only 48.9% knew that drugs could also cause adverse effects. However, interestingly it was seen that 71.6% of the participants knew that unnecessary drug use during pregnancy can harm the health of mother and fetus but they did not significantly know about the nature of harm as only 30.6% knew that drugs could also harm fetal organogenesis and development. A study in Ethiopia also shows that knowledge of pregnant women on NSAIDs (Non-Steroidal Anti-Inflamatory Drugs) was poor [5] and another study in India shows that only 33.33% of the pregnant women had knowledge that drug use during pregnancy might impact the health of mother and child [20]. This study also shows a significant association between KAP scores with knowledge and occupation of the respondants. With increasing education, patients possessed better KAP towards pregnancy. Another similar study has reported a significant association (p = 0.009) between level of education and practice of folic acid intake during pregnancy [21].

Remarkable percentage of pregnant women in this study have shown positive attitude towards medication use during pregnancy. More than 75.6% of the participants replied that they should have asked about their complications and medicines with physician or pharmacist. Patients also had a habit of self medication (64.2%), but majority stopped such habit when they knew that they were pregnant. A higher percentage has been reported in a study in Nigeria where 72.4% of pregnant participants were found to have the habit of self medication [7]. Likewise another study also suggests that use of OTC medications was quite common during pregnancy and more interventional strategies are required to educate patients on this regard [22]. This also emphasizes the need of counseling or other suitable interventions to impart proper knowledge and change the attitude and practice of pregnant women towards medication use during pregnancy. Similar conclusions for effective interventions have also been derived from other studies [5–7]. A study also shows that pharmaceutical labeling was effective to impart warning messages about teratogenicity [18].

The present research found that counseling had a better impact on knowledge, attitude and practice of the pregnant women regarding medication use in pregnancy. Another similar study showed that there was a positive trend between preconception counseling and patient awareness regarding the risks of early pregnancy hyperglycemia [16]. Similarly, in another study, pregnant women who received periconception counseling had better pregnancy outcomes [17].

Since appropriate counseling has been shown to improve medication adherence by the pregnant women [23], we believe that they should be counseled and educated regarding safe and effective medication. Hospitals and other health care facilities should allocate sufficient resources to counsel pregnant women. This would not only improve patient compliance and medication adherence, but could also be a milestone to prevent medication misadventures during pregnancy.

### Limitations of the study

The study was limited to a single hospital and specific geographic area of Nepal. The sample size thus might not be considered sufficient so as to generalize the findings of the study to be representative of total Nepalese pregnant women.

## Conclusion

It was seen that pregnant women possessed inadequate knowledge about their medications. Though they demonstrated relatively better attitude, the practice of medication habits during pregnancy was also not satisfactory. Knowledge, attitudes and practices of pregnant women towards medication use during pregnancy was improved by counseling. Hence, counseling could be an effective intervention to for improving knowledge, attitude and practice outcomes.

## Additional files

Additional file 1: Questionnaires in English (contains the KAP questionnaires which were used in the study). (DOCX 14 kb)

Additional file 2: Participant’s responses to individual questions before and after counseling (contains the number/percentage responses of the participants to all the KAP questions before and after counseling). (DOCX 18 kb)

## Abbreviations

GNI: Gross National Income
KAP: Knowledge, attitude and practice
NSAIDs: Non-steroidal anti-inflamatory drugs
OBG: Obstetrics and gynecology
OPD: Out-patient department
OTC: Over the counter
