# Supplementary material for: Impacts of counseling on knowledge, attitude and practice of medication use during pregnancy
Source: BMC Pregnancy Childbirth. 2017 Apr 27;17:131. doi: 10.1186/s12884-017-1316-6 (PMC5408448; doi:10.1186/s12884-017-1316-6)
Supplement: Supplementary file 1 — Questionnaires in English (contains the KAP questionnaires which were used in the study). (DOCX 14 kb) [file 12884_2017_1316_MOESM1_ESM.docx]

## Additional File 1: Questionnaires in English

**KNOWLEDGE QUESTIONS**

*Please tick on what your appropriate response is regarding the following:*

| **S.N.** | **Question** | **Yes** | **No** | **Uncertain** |
| --- | --- | --- | --- | --- |
| 1. | Do you know about your present complication? |  |  |  |
| 2. | Do you know the name of the medicine that has been prescribed to you? |  |  |  |
| 3. | Do you know the use of all the medicines that you are currently taking? |  |  |  |
| 4. | Do you know that medicines can also show adverse effects? |  |  |  |
| 5. | Do you know that drugs that we take might not be safe in pregnancy? |  |  |  |
| 6. | Do you know that unnecessary drugs taken by the pregnant mother can show adverse effects on the health of the mother and fetus? |  |  |  |
| 7. | Do you know that exposure to unnecessary drugs during pregnancy can affect fetal organogenesis and development? |  |  |  |

**ATTITUDE QUESTIONS**

*Please select the number according to your opinion.*

**1= strongly agree, 2= agree, 3= uncertain, 4= disagree, 5= strongly disagree**

| **S.N.** | **Questions** | **1** | **2** | **3** | **4** | **5** |
| --- | --- | --- | --- | --- | --- | --- |
| 1. | I should ask about my complication and safety of medication during pregnancy with my physician or pharmacist |  |  |  |  |  |
| 2. | I should immediately notify my physician, pharmacist or nurse if any adverse drug reaction is seen |  |  |  |  |  |
| 3. | I should stop taking unnecessary OTC medicines during pregnancy |  |  |  |  |  |
| 4. | Asking about safety of medicines can help prevent unwanted risks |  |  |  |  |  |

**PRACTICE QUESTIONS**

*Please select the number according to your opinion.*

**1= strongly agree, 2= agree, 3= uncertain, 4= disagree, 5= strongly disagree**

| **S.N.** | **Questions** | **1** | **2** | **3** | **4** | **5** |
| --- | --- | --- | --- | --- | --- | --- |
| 1. | I take OTC medications quite frequently without consultation with physician or pharmacist. |  |  |  |  |  |
| 2. | My medicine taking way and habit has changed after knowing that I am pregnant |  |  |  |  |  |
| 3. | For any medicine I am taking (OTC or prescribed), I try to find out whether it is safe during pregnancy or not. |  |  |  |  |  |
| 4. | I am following the instructions provided regarding proper medicine use |  |  |  |  |  |
