# Supplementary material for: Impacts of counseling on knowledge, attitude and practice of medication use during pregnancy
Source: BMC Pregnancy Childbirth. 2017 Apr 27;17:131. doi: 10.1186/s12884-017-1316-6 (PMC5408448; doi:10.1186/s12884-017-1316-6)
Supplement: Supplementary file 2 — Participant’s responses to individual questions before and after counseling (contains the number/percentage responses of the participants to all the KAP questions before and after counseling). (DOCX 18 kb) [file 12884_2017_1316_MOESM2_ESM.docx]

**Additional File 2: Participant’s responses to individual questions before and after counseling**

**Knowledge Questions**

|  |  | **Before Counseling [No. (%)]** | | | **After Counseling [No. (%)]** | | |
| --- | --- | --- | --- | --- | --- | --- | --- |
| **S.N.** | **Question** | **Yes** | **No** | **Uncertain** | **Yes** | **No** | **Uncertain** |
| 1. | Do you know about your present complication? | 156  (68.1) | 17  (7.4) | 56  (24.5) | 209  (91.3) | 0 | 20  (8.7) |
| 2. | Do you know the name of the medicine that has been prescribed to you? | 106  (46.3) | 67  (29.3) | 56  (24.5) | 160  (69.9) | 7  (3.1) | 62  (27.1) |
| 3. | Do you know the use of all the medicines that you are currently taking? | 50  (21.8) | 94  (41.0) | 85  (37.1) | 106  (46.3) | 10  (4.4) | 113  (49.3) |
| 4. | Do you know that medicines can also show adverse effects? | 112  (48.9) | 52  (22.7) | 65  (28.4) | 213  (93.0) | 0 | 16  (7.0) |
| 5. | Do you know that drugs that we take might not be safe in pregnancy? | 151  (65.9) | 42  (18.3) | 36  (15.7) | 229  (100) | 0 | 0 |
| 6. | Do you know that unnecessary drugs taken by the pregnant mother can show adverse effects on the health of the mother and fetus? | 164  (71.6) | 41  (17.9) | 24  (10.5) | 227  (99.1) | 0 | 2  (0.9) |
| 7. | Do you know that exposure to unnecessary drugs during pregnancy can affect fetal organogenesis and development? | 70  (30.6) | 83  (36.2) | 76  (33.2) | 219  (95.6) | 2  (0.9) | 8  (3.5) |

**Attitude questions**

**1= strongly agree, 2= agree, 3= uncertain, 4= disagree, 5= strongly disagree**

|  |  | **Before Counseling [No. (%)]** | | | | | **After Counseling [No. (%)]** | | | | |
| --- | --- | --- | --- | --- | --- | --- | --- | --- | --- | --- | --- |
| **S.N.** | **Questions** | **1** | **2** | **3** | **4** | **5** | **1** | **2** | **3** | **4** | **5** |
| 1. | I should ask about my complication and safety of medication during pregnancy with my physician or pharmacist | 16 (7) | 157 (68.6) | 52 (22.7) | 2 (0.9) | 2 (0.9) | 76 (33.2) | 139 (60.7) | 14 (6.1) | - | - |
| 2. | I should immediately notify my physician, pharmacist or nurse if any adverse drug reaction is seen | 21  (9.2) | 133  (58.1) | 74  (32.3) | 1  (0.4) | - | 81  (35.4) | 137  (59.8) | 11  (4.8) | - | - |
| 3. | I should stop taking unnecessary OTC medicines during pregnancy | 21  (9.2) | 121  (52.8) | 83  (36.2) | 4  (1.7) | - | 164  (71.6) | 62  (27.1) | 3  (1.3) | - | - |
| 4. | Asking about safety of medicines can help prevent unwanted risks | 34  (14.8) | 140  (61.1) | 54  (23.6) | 1  (0.4) | - | 128  (55.9) | 95  (41.5) | 6  (2.6) | - | - |

**Practice questions**

**1= strongly agree, 2= agree, 3= uncertain, 4= disagree, 5= strongly disagree**

|  |  | **Before Counseling [No. (%)]** | | | | | **After Counseling [No. (%)]** | | | | |
| --- | --- | --- | --- | --- | --- | --- | --- | --- | --- | --- | --- |
| **S.N.** | **Questions** | **1** | **2** | **3** | **4** | **5** | **1** | **2** | **3** | **4** | **5** |
| 1. | I take OTC medications quite frequently without consultation with physician or pharmacist. | 27  (11.8) | 120  (52.4) | 13  (5.7) | 64  (27.9) | 5  (2.2) | - | 5  (2.2) | 35  (15.3) | 138  (60.3) | 51  (22.3) |
| 2. | My medicine taking way and habit has changed after knowing that I am pregnant | 36  (15.7) | 137  (59.8) | 29  (12.7) | 25  (10.9) | 2  (0.9) | 138  (60.3) | 77  (33.6) | 12  (5.2) | 2  (0.9) | - |
| 3. | For any medicine I am taking (OTC or prescribed) , I try to find out whether it is safe during pregnancy or not. | 2  (0.9) | 23  (10.0) | 37  (16.2) | 98  (42.8) | 69  (30.1) | 11  (4.8) | 47  (20.5) | 108  (47.2) | 58  (25.3) | 5  (2.2) |
| 4. | I am following the instructions provided regarding proper medicine use | 24  (10.5) | 101  (44.1) | 50  (21.8) | 49  (21.4) | 5  (2.2) | 125  (54.6) | 78  (34.1) | 20  (8.7) | 6  (2.6) | - |
